# A tetrahedron from homooxacalix[3]arene, the fifth Platonic polyhedron from calixarenes and uranyl

**DOI:** 10.3389/fchem.2023.1163178

**Published:** 2023-04-21

**Authors:** Jin-Cheng Wu, Eduardo C. Escudero-Adán, Marta Martínez-Belmonte, Javier de Mendoza

**Affiliations:** The Institute of Chemical Research of Catalonia (ICIQ), Tarragona, Spain

**Keywords:** uranyl–organic framework, metallo-cages, self-assembly, calixarenes, porous materials

## Abstract

A self-assembled tetrahedral cage results from two *C*_3_-symmetry building blocks, namely, homooxacalix[3]arene tricarboxylate and uranyl cation, as demonstrated by X-ray crystallography. In the cage, four metals coordinate at the lower rim with the phenolic and ether oxygen atoms to shape the macrocycle with appropriate dihedral angles for tetrahedron formation, whereas four additional uranyl cations further coordinate at the upper-rim carboxylates to finalize the assembly. Counterions dictate the filling and porosity of the aggregates, whereas potassium induces highly porous structures, and tetrabutylammonium yields compact, densely packed frameworks. The tetrahedron metallo-cage complements our previous report (Pasquale et al., Nat. Commun., 2012, 3, 785) on uranyl–organic frameworks (UOFs) from calix[4]arene and calix[5]arene carboxylates (octahedral/cubic and icosahedral/dodecahedral giant cages, respectively) and completes the assembly of all five Platonic solids from just two chemical components.

## Introduction

Metals have been key players since the birth of supramolecular chemistry, not only for their role as templates in the generation of macrocyclic compounds of different shapes and sizes but also for producing and being incorporated into a wide range of self-assembled structural motifs such as helicates or grids of increasing complexity and controlled topologies (i.e., molecules whose representation/graph based on atoms and bonds is non-planar), like catenanes, rotaxanes, knots, molecular muscles, and machines, many of them pioneered by Prof. Jean-Pierre Sauvage and thoroughly described in his Nobel Lecture (Sauvage, 2017). In the context of metallosupramolecular chemistry, metal-mediated self-assembled spheres or polyhedral cages have gained increased attention, owing to their nanoscale cavities. Work in this area has mostly focused on building their architectures and studying the properties and applications of their confined nanospaces. The topic has been extensively reviewed over the last four decades, and some recent studies include Pullen et al. (2021), McConnell (2022), and McTernan et al. (2022).

Design principles to construct high-symmetry cages, such as tetrahedra, cubes, and octahedra, were first discussed by Stang and co-workers (Chakrabarty et al., 2011) and are based on the shapes of the ligands employed and the coordination angles of the metals involved. The ample choice of available metal candidates for self-assembly must be balanced by the rigidity of their coordination requirements, much higher than other tools such as hydrogen bonds or hydrophobic forces. For example, in Fujita’s M_*n*_L_*2n*_ (M = Pd^2+^) giant nanoscopic metallo-cages (Sun et al., 2011), subtle variations in the ligand (furan *versus* thiophene) favor either M_12_L_24_ or M_24_L_48_ rhombicuboctahedral constructs. In addition, the self-assembly process must be reversible to reach equilibrium, so the most stable structures result. Thus, kinetically labile octahedral (i.e., GaIII, FeII, CoII, ZnII, and NiII) (Caulder and Raymond, 1999) and square planar (PdII and PtII) metal ions (Sun et al., 2020) are usually employed. Among the larger metals, lanthanides (i.e., EuIII) have also been used for self-assembly, but their variable coordination numbers and geometries complicate rational designs (Yan et al., 2015). In the actinide series, uranyl cation UO_2_^2+^ has been employed to assemble frameworks and cages, but most examples generally involve polyoxometalate-type clusters (Burns et al., 2005; Ling et al., 2010; Thuéry and Harrowfield, 2017).

Among the self-assembled cages, the chemical replica of Platonic solids, the five regular polyhedra with convex faces, have always attracted and fascinated synthetic chemists. While polyhedral structures based on sp^3^-carbon atoms are limited to tetrahedrane, cubane, and dodecahedrane, whose syntheses constituted milestone achievements decades ago (Eaton and Cole, 1964; Maier et al., 1978; Ternansky et al., 1982), coordination and supramolecular chemistry have opened access to structures of increased complexity (Seidel and Stang, 2002; Pluth and Raymond, 2007; Yoshizawa et al., 2009; Hardie, 2010; Jin et al., 2010; Smulders et al., 2013; Young and Hay, 2013), such as octahedra (MacGillivray and Atwood, 1997; Takeda et al., 1999; Ronson et al., 2007; Hiraoka et al., 2009), icosahedra (Orr et al., 1999; Bilbeisi et al., 2013), tetrahedra (Pluth et al., 2007; Mal et al., 2009; Granzhan et al., 2011; Clustelcean et al., 2012; Mahata et al., 2013; Mitra et al., 2013), or so-called Archimedean solids (Olenyuk et al., 1999; Sun et al., 2010; Liu et al., 2011; Wang et al., 2014).

**FIGURE 1:**
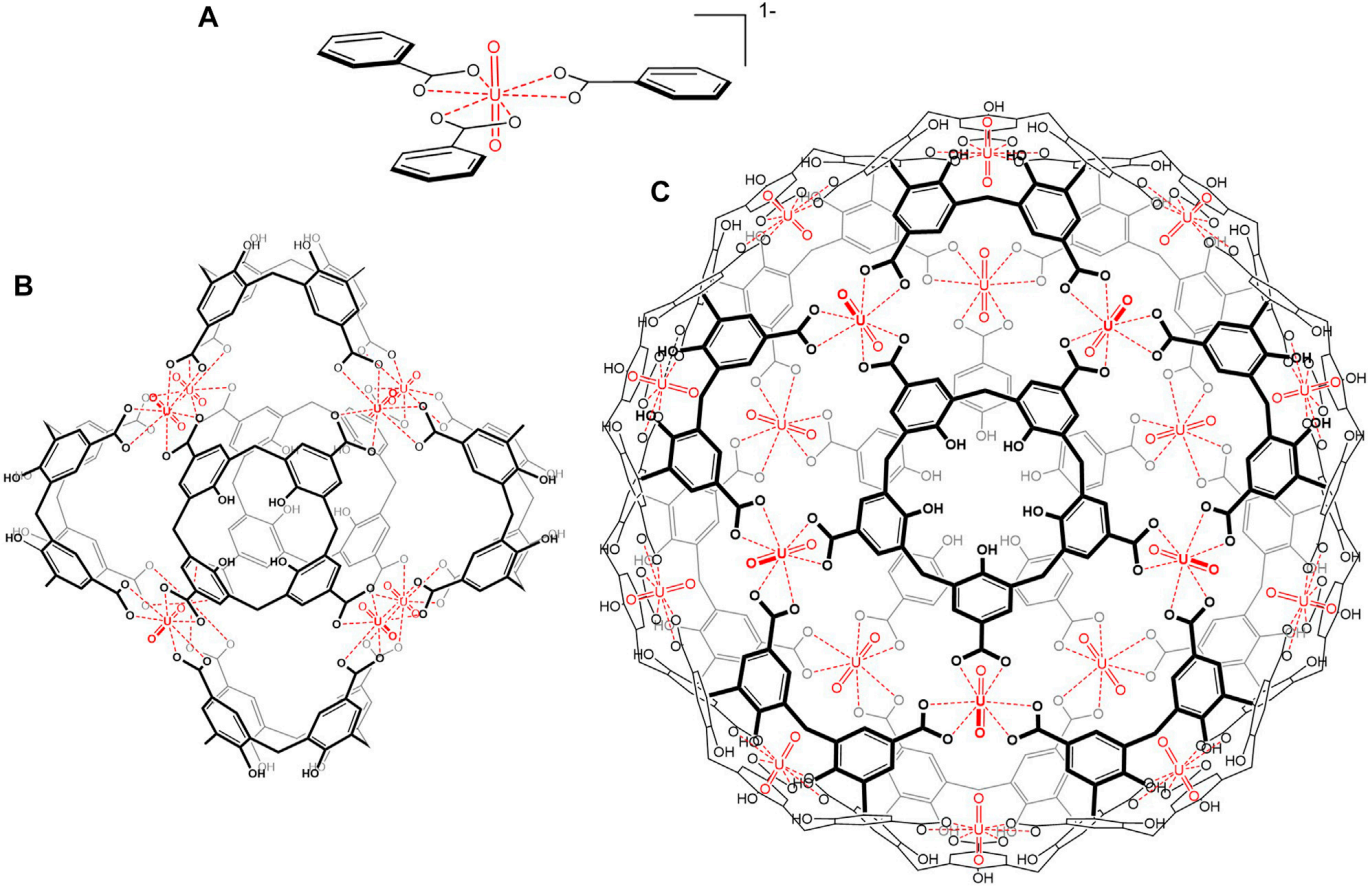
Hexagonal bipyramidal coordination of uranyl with carboxylates **(A)** and chemical structures of uranyl hexameric **(B)** and dodecameric **(C)** cages from calixarenes.

We described, a decade ago, novel metallo-cages in the solid state arising from uranyl cation UO_2_^2+^ and calixarene carboxylic acids (Pasquale et al., 2012). Uranyl easily coordinates reversibly with three carboxylates at its equatorial plane in a hexagonal bipyramidal fashion (Clark et al., 1995; Sather et al., 2010; Wang and Chen, 2011), providing an ideal *C*_3_-symmetry component (Figure 1A), whereas calix[4]arene and calix[5]arene carboxylates provide *C*_4_- and *C*_5_-symmetry elements, respectively, to build octahedral and icosahedral assemblies (with an inner cube or dodecahedron inscribed at the uranyl -*yl* oxygens, respectively) (Figures 1B, C). Indeed, polycarboxylates have been often employed as ligands for uranyl–organic frameworks (UOFs) (Thuéry et al., 1999; Thuéry et al., 2004; Liao et al., 2010; Wang and Chen, 2011; Li et al., 2016; Zhang et al., 2017; Hu et al., 2018) with a wide variety of resulting architectures.

As a result, octahedral and icosahedral anionic metallo-cages of nanoscopic dimensions (estimated inner volumes from inscribed spheres *ca*. 940 and 7,200 Å^3^, respectively) were formed univocally with an unusually small number of components (Pasquale et al., 2012).

The assembly of a tetrahedron, the last Platonic solid from calixarene carboxylic acids and uranyl, requires two *C*_3_-symmetry components in a L_4_M_4_ stoichiometry, but the selection of an appropriate ligand with three carboxylates is by no means trivial. Calix[6]arenes substituted at alternate rings are unsuitable candidates, since these substituents are oriented almost parallel to each other in their conformations (van Duynhoven et al., 1994). An interesting alternative would be the use of *O*-unsubstituted homooxacalix[3]arenes, but they display wide, almost flat cone conformations (Tsubaki et al., 1998). Interestingly, however, the cavities can shrink upon uranyl coordination with the six oxygen atoms of the macrocycle (three phenols and three ether bridges) (Thuéry et al., 1999; Masci et al., 2002) into ideal angles and shapes for a tetrahedral assembly.

**SCHEME 1:**
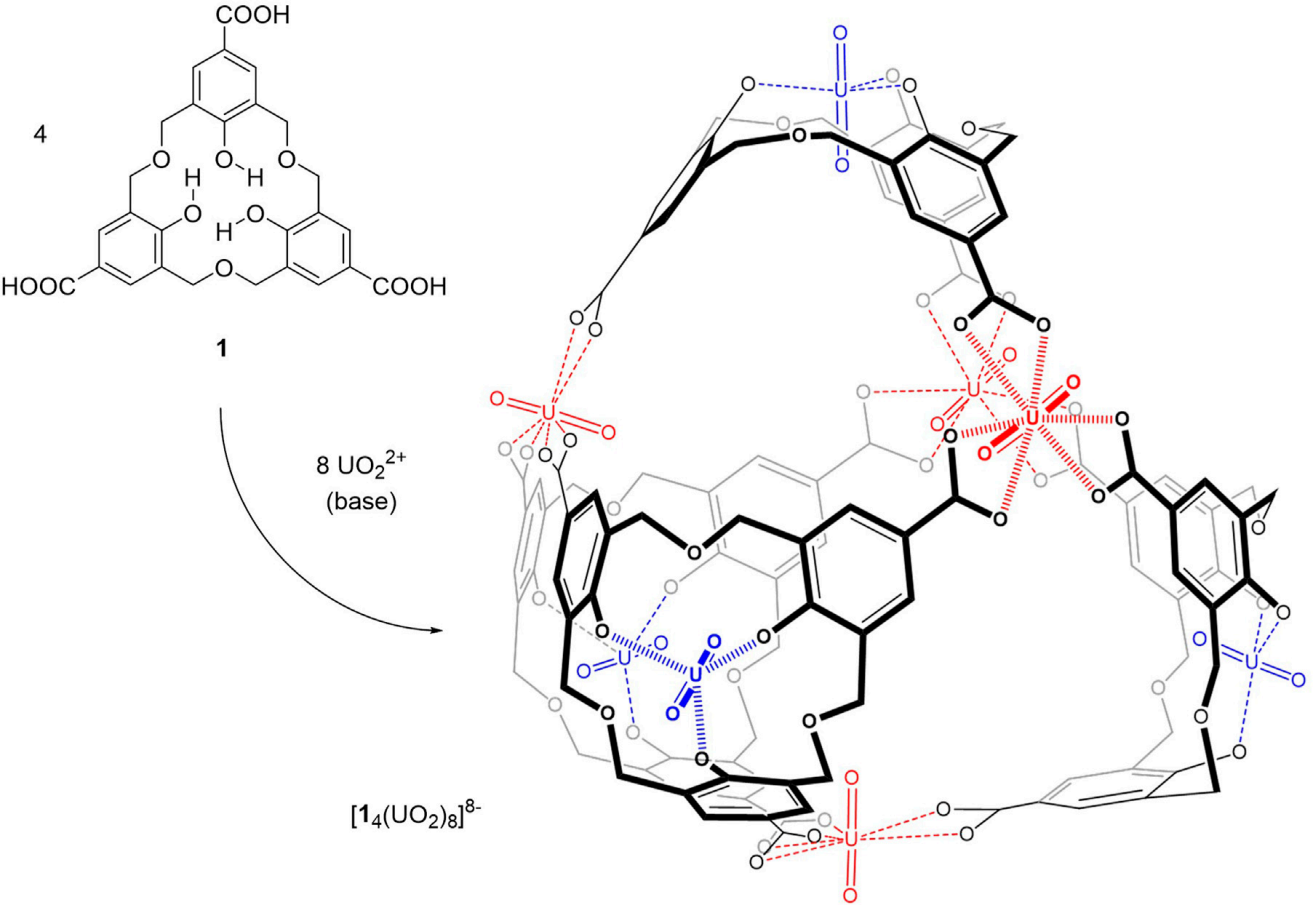
Assembly of a tetrahedral cage from homooxacalix[3]arene tricarboxylic acid (**1**) and uranyl.

Based on these findings, a cage of L_4_M_8_ stoichiometry could be anticipated from homooxacalix[3]arene tricarboxylic acid 1 (Scheme 1), with four uranyl cations (in blue) at the lower rim of the ligands (shaping metals) and the other four metals (in red) acting as gluing elements.

## Results and discussion

**FIGURE 2:**
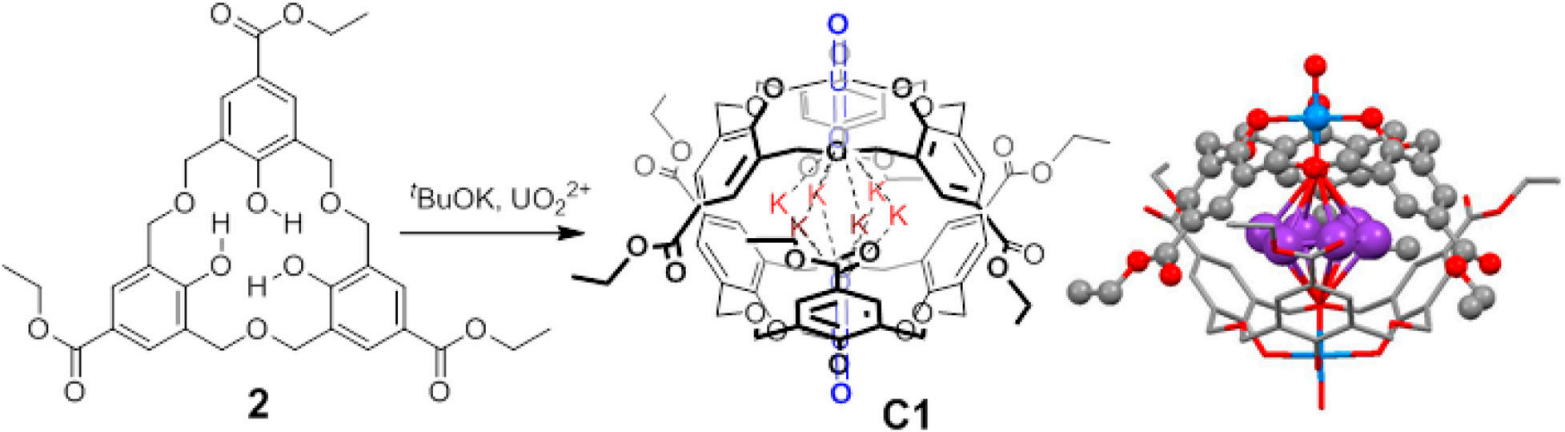
Synthesis and crystal structure of uranyl complex **C1** from triethyl homooxacalix[3]arene tricarboxylate ester (**2**).

The predicted shaping of homooxacalix[3]arene–uranyl complexes into an ideal building block for tetrahedron formation was confirmed in the solid state from the triester precursor of triacid **1**, namely, triethyl homooxacalix[3]arene tricarboxylate (**2**) (Zhong et al., 1999). Indeed, a single crystal (crystal **C1**) was grown from the slow diffusion of ethyl acetate into a mixture of **2**, potassium *tert*-butoxide, and uranyl nitrate in a CHCl_3_–methanol–DMF solvent mixture. In the crystal, the uranyl is bound to all three phenol groups of the macrocycle, at the expected long U-O single-bond distances (*ca*. 2.22 Å) (Thuéry et al., 1999), forcing the calixarene skeleton to adopt a sharp conical bowl-shaped conformation (dihedral angles of benzene rings at *ca.* 85.3^o^) (Figure 2; Supplementary Figure S1). The remaining ether oxygen atoms of the macrocycle also coordinate at the uranyl equatorial plane, though in a much weaker manner (U-O distances *ca.* 3.19 Å).

Interestingly, in the crystal packing, **C1** forms a dimeric capsule, arising from two staggered, face-to-face oriented bowls and stabilized by the disordered potassium counterions of both units, bound to the inner -*yl* oxygen atoms of the uranyl moieties, and also stabilized by cation–*π* interactions with the neighboring benzene rings (Gokel et al., 2002).

Tricarboxylic acid **1** was readily synthesized by the hydrolysis of **2** with potassium hydroxide in an ethanol–water mixture. Slow diffusion of toluene into a solution of **1**, potassium hydroxide, and uranyl nitrate in DMF resulted in a crystal showing the expected supramolecular assembly. The structure was resolved using a rotating anode with MoK_*a*_ radiation, without requiring the use of a synchrotron light beam, as is usual for most giant assemblies (Sun et al., 2010; Sun et al., 2011).

**FIGURE 3:**
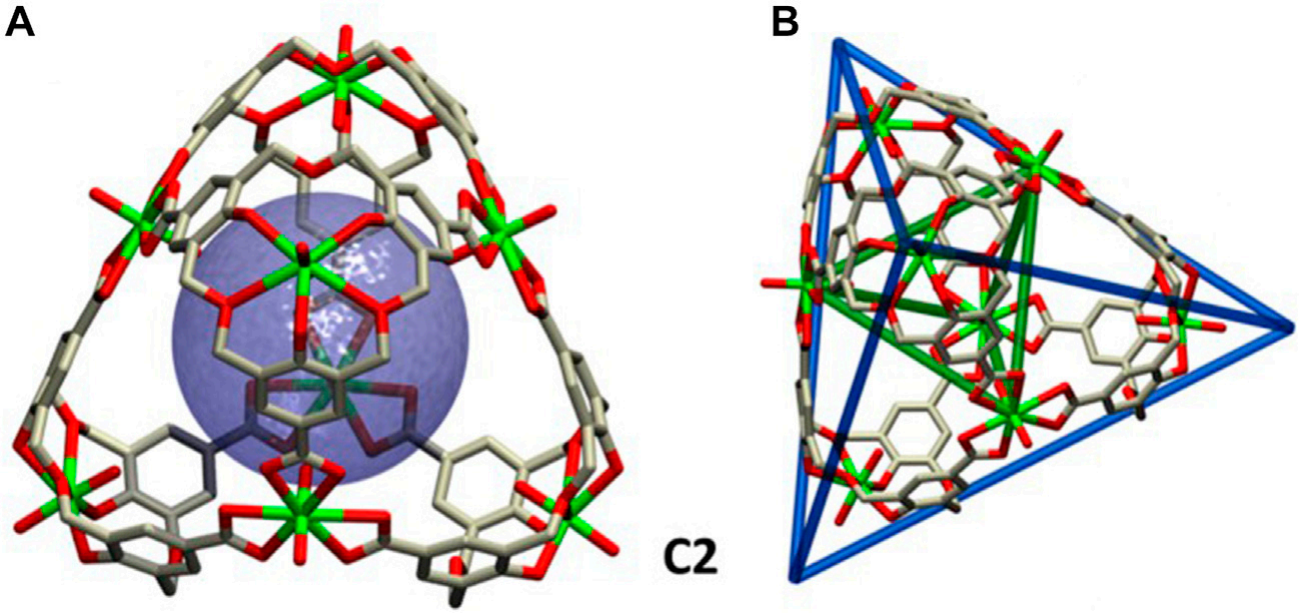
Crystal structure **C2**. **(A)** Tetrahedron frame with a *ca.* 382 Å^3^ sphere tangent to the -*yl* oxygens at the faces, representing the inner available volume. **(B)** Wireframe representation of the two distorted tetrahedrons defined by the inner -*yl* uranyl oxygens on the face (green lines) and by the outer tetrahedral surface (blue lines).

The tetrahedral complex **1**_4_(UO_2_)_8_K_8_ (crystal **C2**) crystallizes in the body-centered cubic space group I-43m, showing a high degree of symmetry. Four uranyl residues are located at the center of the faces, whereas the remaining four metals lie at the corners (Figure 3). Unlike for crystal **C1**, the calixarene monomers are not fully symmetric, revealing a certain degree of distortion, as only two of the ether oxygen atoms bind to the uranyl (Supplementary Figure S2B). The inner -*yl* oxygens of the uranyl groups at the faces define a tetrahedron with two *ca*. 7.70 Å edges and four *ca*. 7.93 Å edges, whereas a larger tetrahedron (Figure 3B; Supplementary Figure S3) is defined by the prolongation of the lines along the edges of the assembly.

**FIGURE 4:**
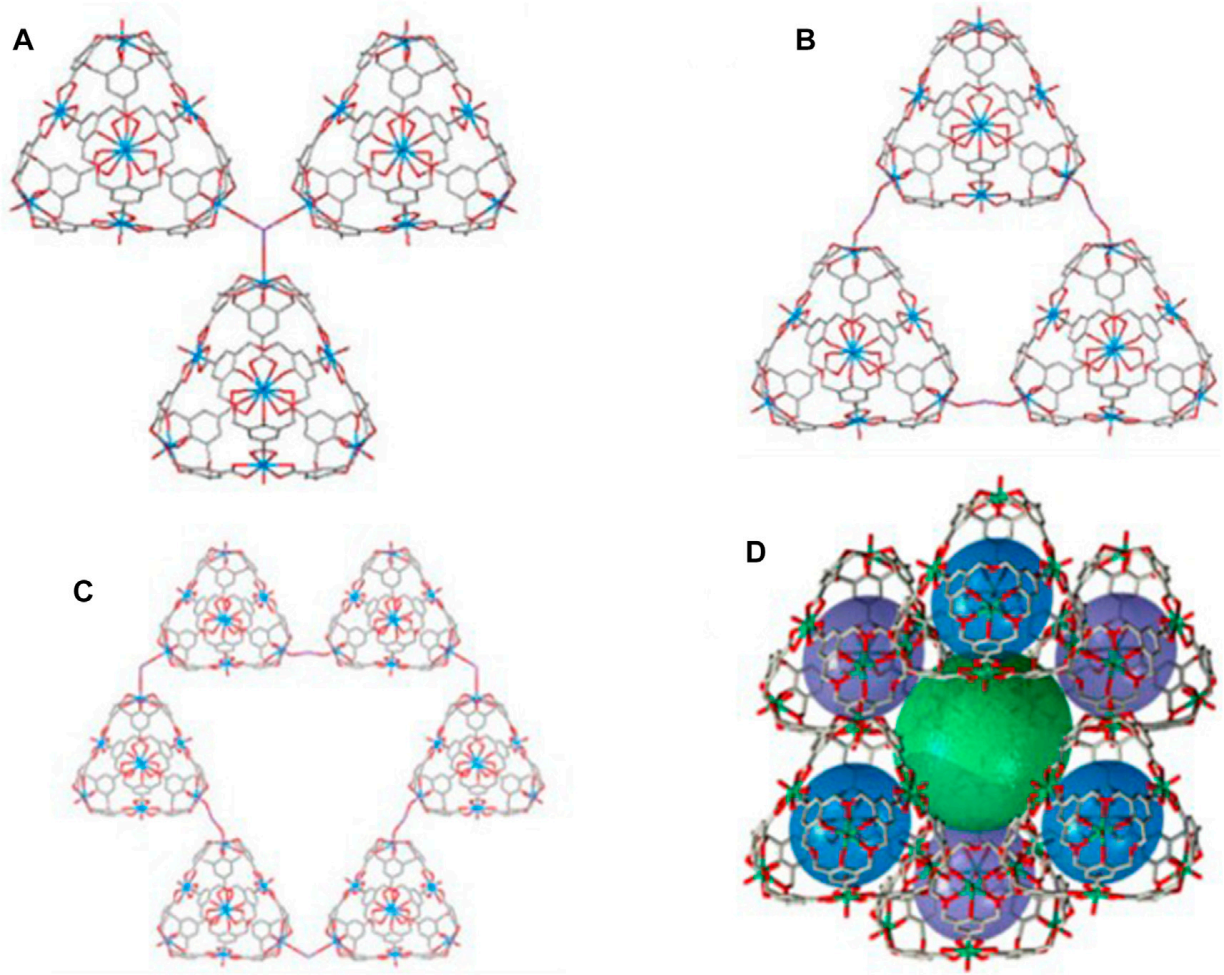
Stacking mode between tetrahedral cages and bridged potassium counterions in **C2**. **(A)** Each potassium bridges three tetrahedral cages. **(B)** Three tetrahedra form a circle *via* three bridged potassium cations. **(C)** Six tetrahedra in a circle *via* six bridged potassium atoms. **(D)** Self-assembled cavity (in green) formed by six tetrahedral cages with a *ca*. 882 Å^3^ volume.

The crystal packing of **C2** reveals a remarkable degree of porosity. Each tetrahedron requires eight cationic counterions to balance the overall negative charge (one negative charge per uranyl subunit). In crystal **C2**, only four disordered potassium cations have been assigned, located as a bridge connecting to three tetrahedra *via* the outer -*yl* uranyl oxygens at the corners (Figure 4A).

The three-dimensional stacking of tetrahedra and potassium counterions in **C2** is based on triple-tetrahedral subunits and a sextuple-tetrahedral subunit formed through potassium bridges (Figures 4A–C). Two triple-tetrahedral subunits are formed: one *via* a bridged potassium atom and the other as a circle that requires three bridged potassium counterions. Also, a larger, flat, cyclic subunit is formed by six tetrahedrons and six bridged potassium atoms. Stacking of both triple-tetrahedral assemblies produces a large tetrahedral cavity, whose potassium vertexes define 19.56 Å edges and an inner available volume of *ca*. 882 Å^3^ (Figure 4D; Supplementary Figure S4). The complexity further increases by the formation of a cage with 12 tetrahedra, in which the sextuple-tetrahedral assembly cage is embedded (Supplementary Figure S5). The 3D tetrahedron–potassium network in **C2** can be displayed by using layer-by-layer stacking and mutually embedding patterns (Supplementary Figure S6).

**FIGURE 5:**
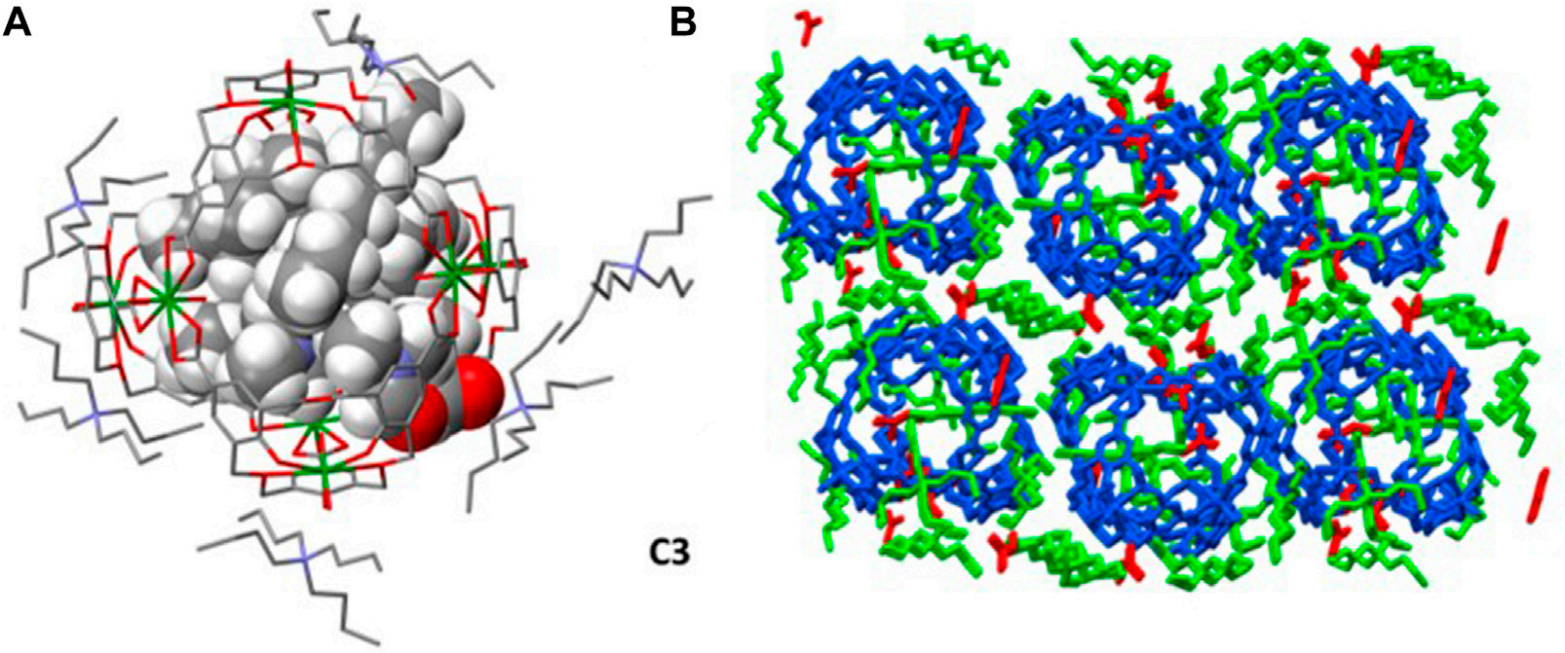
**(A)** Two TBAs and three DMFs filling the tetrahedral cage, whereas six TBAs surround the cage in **C3**. **(B)** Packing of tetrahedral cages, TBA counterion, and DMF solvent molecules.

When ethyl acetate is diffused into a mixture of **1**, tetrabutylammonium (TBA) hydroxide, and uranyl nitrate, a homooxacalix[3]arene–uranyl tetrahedral complex (crystal **C3**) is also formed. The complex with TBA as a counterion crystalizes in space group P2 (1)/n, a lower symmetry than crystal **C2**, so that the tetrahedral cage lacks symmetry elements. In this case, all eight cationic TBA counterions are located. Two of them, together with three solvent molecules (DMF), fulfill the cavity (Figure 5A). The remaining six TBA counterions surround the tetrahedral cage in a tight packing, so a porous assembly is not present under these conditions (Figure 5B). This is rather unusual in other chemical replica of Platonic tetrahedra (Granzhan et al., 2011; Clustelcean et al., 2012).

## Summary and outlook

In summary, all five Platonic solids can be easily assembled from just two components, namely, calixarene carboxylates and uranyl. The last one, the tetrahedron (representing fire in Plato’s conception of world), is described here. The assembly requires homooxacalix[3]arene tricarboxylate and eight uranyl moieties, four of them employed to shape the macrocycle into the appropriate conformation, while the remaining four are gluing elements to bridge the subunits by carboxylate–uranyl coordination. Counterions dictate the packing characteristics. Potassium creates porous materials, whereas tetrabutylammonium yields densely packed structures. As for the remaining uranyl cages, metals are centered in the faces, paving the way for the use of these novel UOFs as catalytic vessels (Hu et al., 2018), gas storage containers (Furukawa et al., 2010; Li et al., 2016), and in photoelectronic applications (Wang and Chen, 2011; Wang et al., 2012; Wang et al., 2013).

From a design point of view, our approach to Platonic polyhedral cages could be conceptually extended to reversible non-metallic motifs, such as imines, from calixarene aldehydes and planar *C*_3_-symmetry counterparts (i.e., benzene or 1,3,5-triazine triamines) (Rue et al., 2011; Lin et al., 2012), opening the way for a new family of self-assembled large capsules.

## Data Availability

The datasets presented in this study can be found in online repositories. The names of the repository/repositories and accession number(s) can be found below: http://www.crystallography.net/cod/-, 3000431, 3000432, and 3000433.
