# Supplementary material for: A tetrahedron from homooxacalix[3]arene, the fifth Platonic polyhedron from calixarenes and uranyl
Source: Front Chem. 2023 Apr 21;11:1163178. doi: 10.3389/fchem.2023.1163178 (PMC10160636; doi:10.3389/fchem.2023.1163178)
Supplement: Supplementary file 1 [file DataSheet1.pdf]

Supplementary Material

A Tetrahedron from Homooxacalix[3]arene, the Fifth Platonic Polyhedron from Calixarenes and Uranyl

Jin-Cheng Wu, Eduardo C. Escudero-Adán, Marta Martínez-Belmonte, and Javier de Mendoza*

The Institute of Chemical Research of Catalonia (ICIQ), Avda. Països Catalans 16, 43007-Tarragona, Spain

Email: jmendoza@iciq.es

Table of Contents

General Methods S1

Synthesis and crystal data S2

X-Ray diffraction analysis for **C1-C3** S3

^{1}H and ^{13}C NMR spectra for **1** S6

General Methods

^{1}H and ^{13}C NMR spectra were recorded on a Bruker AV-400 (300 MHz) spectrometer. All NMR data were collected at 300 K unless otherwise specified and chemical shifts were reported as the delta scale in ppm. All HR mass spectra were recorded on a Maldi TOF Bruker Autoflex. Single-crystal X-ray diffraction data were collected on a Bruker FR591 rotating anode equipped with Montel mirrors with MoKα radiation (λ = 0.71073 Å) and a Bruker Apex-II Duo diffractometer equipped with micro sources of CuKα (λ = 1.54178 Å) and MoKα radiation (λ = 0.71073 Å), and cooled with an Oxford Cryostream low-temperature device [T = 100(2) K].

Synthesis and Crystal Data

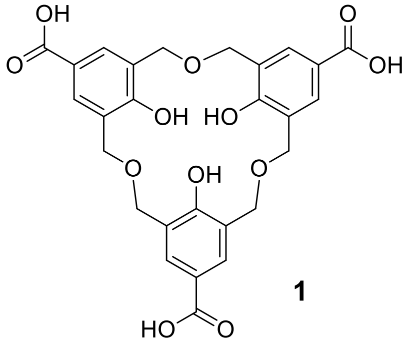

1

A mixture of triethyl homooxacalix[3]arene tricarboxylate (**2**) (Zhong at al. 1999) (125 mg, 0.2 mmol) and KOH (561 mg, 10 mmol) in ethanol (30 mL) and water (30 mL) was heated to reflux for 24 hours. After cooling, 1N HCl (10 mL, 10 mmol) was added to the mixture, and a colorless solid was formed. Homooxacalix[3]arene tricarboxylic acid (**1**) was collected by filtration, washed by ethanol, and dried to give a colorless solid (95 mg, 0.176 mmol) in 88 % yield. Mp: > 360 °C. IR (KBr) ν 3329, 1681, 1605, 1190 cm^{-1}. ^{1}H NMR (300 MHz, DMSO-d_6): δ 12.61 (br, 3H), 9.18 (s, 3H), 7.85 (s, 6H), 4.73 (s, 12H). ^{13}C NMR (125 MHz, DMSO-d_6): δ 166.8, 159.3, 131.6, 124.2, 121.5, 68.9; HRMS-MALDI (*m/z*) $[M-H]^-$ calc. for $C_{27}H_{23}O_{12}$ = 539.1195, found = 539.1170 (4.6 ppm).

Single Crystal Growth:

C1:
A solution of **2** (1.00 mg, 0.0016 mmol), in $CHCl_3$ (0.5 mL) was mixed with 0.342 mL of a solution of potassium *tert*-butoxide (0.54 mg, 0.0048 mmol) in methanol (0.342 mL) and a solution of $UO_2(NO_3)_2$•$6H_2O$ (0.80 mg, 0.0016 mmol), in DMF (0.167 mL). Ethyl acetate was diffused into this mixture, and a green crystal which suitable for single crystal x-ray diffraction was obtained after one week.

C2:
A mixture of **1** (7.8 mg, 0.015 mmol) and KOH (5.19 mg, 0.093 mmol) in DMF (3 mL) was stirred for 3 hours. 0.5 mL of the resulting solution was mixed with a solution of $UO_2(NO_3)_2$•$6H_2O$ (2.51 mg, 0.005 mmol) in DMF (0.420 mL). Toluene was diffused into the mixture to grow the crystal. Green crystals suitable for X-ray single crystal diffraction were obtained after two weeks.

C3:
A mixture of **1** (8.1 mg, 0.016 mmol) in DMF (9.04 mL) and tetrabutylammonium hydroxide (24.91 mg, 0.096 mmol) in methanol (0.960 mL) was kept overnight under stirring. 1 mL of the resulting solution was mixed with a solution of $UO_2(NO_3)_2$•$6H_2O$ (1.61 mg, 0.0032 mmol) in DMF (0.270 mL). Ethyl acetate was diffused into the mixture to grow the crystal. After ten days green crystals suitable for X-ray single crystal diffraction were collected.

X-Ray Diffraction Analysis for C1-C3

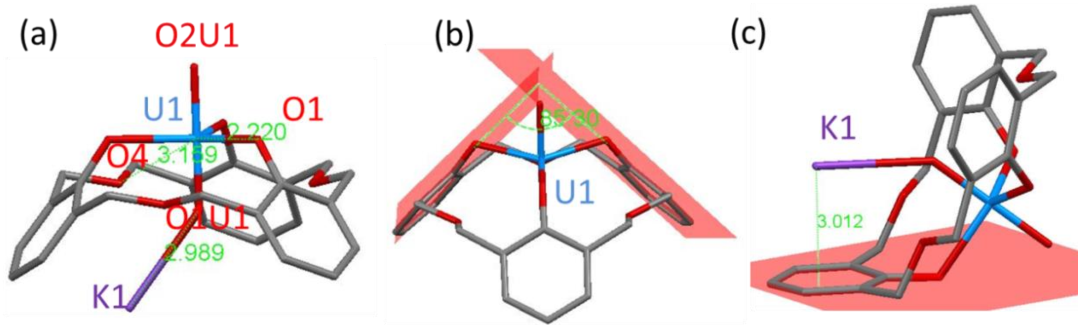

Figure S1. Selected distances and angles in **C1**. (**a**) 2.220 Å (O1-U1), 3.169 Å (O4-U1), 2.989 Å (O1U1-K1). (**b**) Dihedral angle between benzene rings 85.30 Å. (**c**) Distance between K^+ counterion and center of benzene ring 3.102 Å.

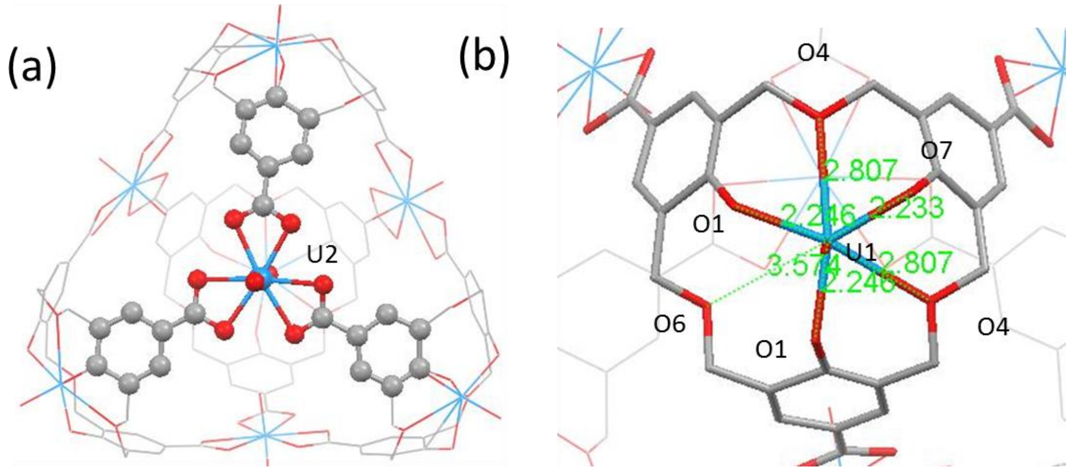

Figure S2. Details of cage **C2**. (**a**) Uranyl cation at the center of the tetrahedron face. (**b**) Uranyl cation at the lower rim of the homooxacalix[3]arene. Distances: 2.246Å (O1-U1), 2.233Å (O7-U1), 2.807Å (O4-U1); 3.574Å (O6-U1, not bonded).

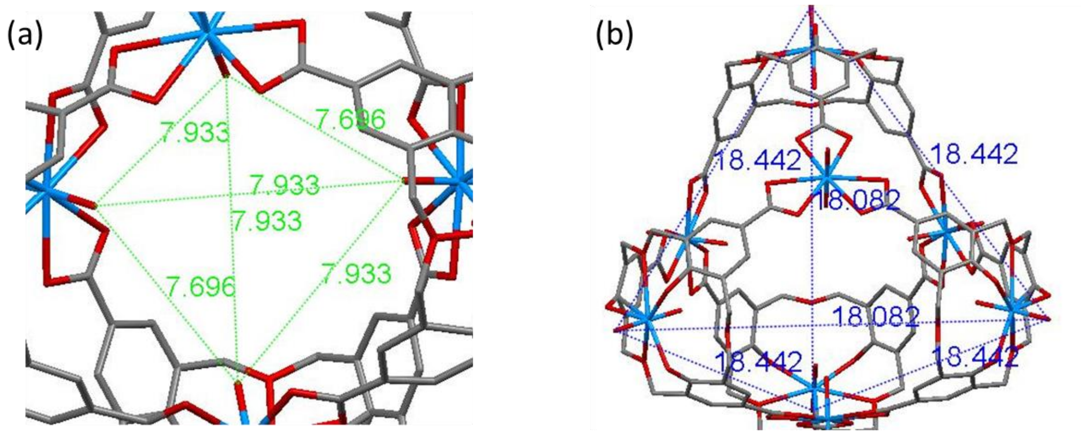

Figure S3. The defined tetrahedral in crystal **C2**: (**a**) Tetrahedron defined by the inner -*yl* oxygens of the uranyl ions at the faces: two 7.696 Å edges and four 7.933 Å edges. (**b**) Tetrahedron defined by the outer -*yl* oxygens of the uranyl ions at the lower rim: two 18.042 Å edges and four 18.442 Å edges.

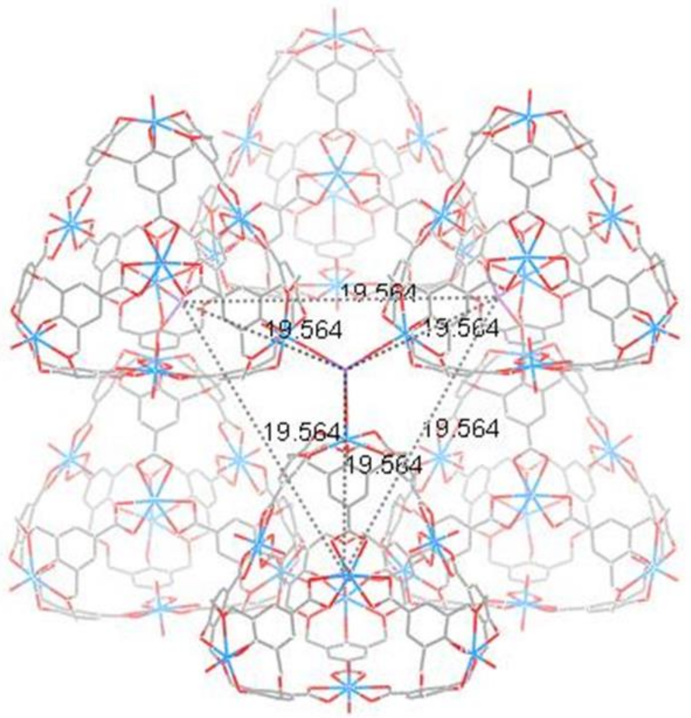

Figure S4. The tetrahedral cavity of *ca* 19.56 Å edges in a sextuple-tetrahedra assembled subunit.

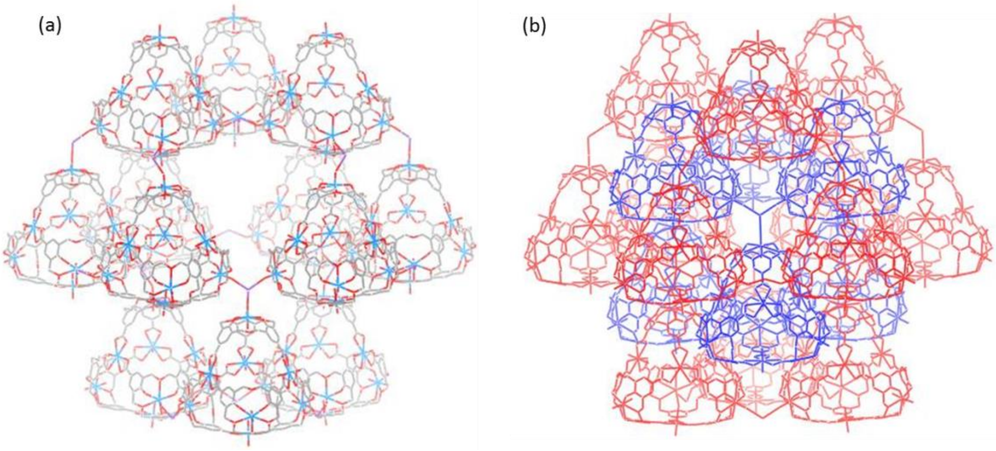

Figure S5. (**a**) A large self-assembled cage subunit composed by twelve tetrahedra. (**b**) The sextuple-tetrahedra self-assembled subunit (in blue) embedded into the large cage (in red).

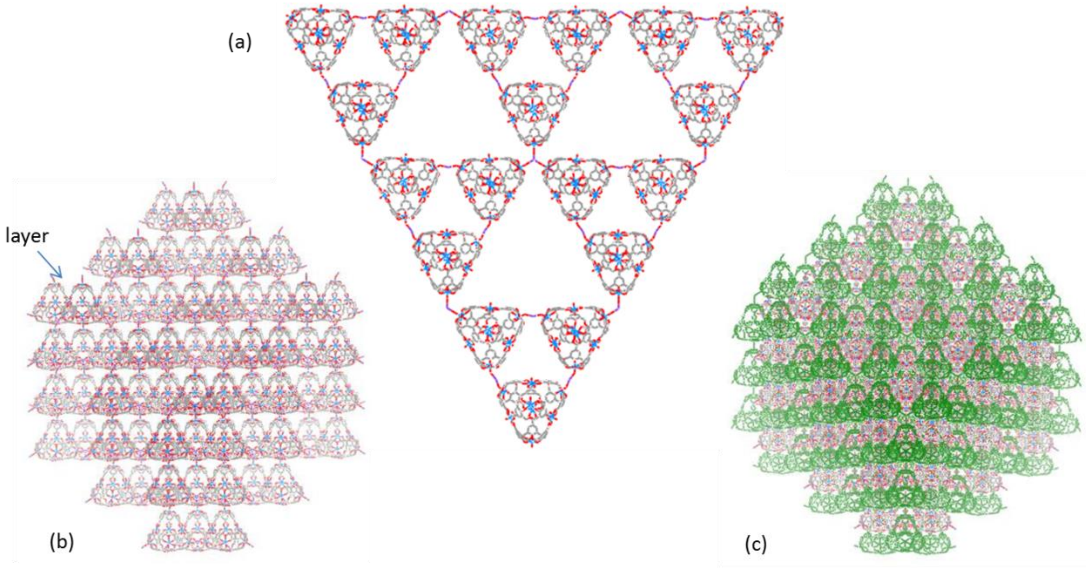

Figure S6. Layer-by-layer stacking of tetrahedra and potassium counterions in **C2**: (**a**) One layer is constructed by triple-tetrahedra subunits and sextuple-tetrahedra subunits. (**b**) The layers are stacked to each other *via* bridged potassium counterions. (**c**) The multiple layers can be embedded into each other to generate a porous network.

Table S1. Crystal data and structure refinement for C1.

Identification code	**C1**	
Empirical formula	C66 H66 K4 O28 U2	
Formula weight	1939.64	
Temperature	100(2) K	
Wavelength	0.71073 Å	
Crystal system	Cubic	
Space group	Ia-3	
Unit cell dimensions	a = 23.722(5) Å	a= 90°.
	b = 23.722(5) Å	b = 90°.
	c = 23.722(5) Å	g = 90°.
Volume	13350(8) $Å^3$	
Z	8	
Density (calculated)	1.930 Mg/m^3	
Absorption coefficient	5.184 mm^{-1}	
F(000)	7568	
Crystal size	0.01 x 0.01 x 0.01 mm^3	
Theta range for data collection	1.717 to 28.307°.	
Index ranges	-15<=h<=31,-18<=k<=31,-15<=l<=31	
Reflections collected	18301	
Independent reflections	2784[R(int) = 0.1369]	
Completeness to theta =28.307°	99.9 %	
Absorption correction	Empirical	
Max. and min. transmission	0.950 and 0.633	
Refinement method	Full-matrix least-squares on F^2	
Data / restraints / parameters	2784/ 6/ 159	
Goodness-of-fit on F2	1.127	
Final R indices [I>2sigma(I)]	R1 = 0.0563, wR2 = 0.1277	
R indices (all data)	R1 = 0.1184, wR2 = 0.1543	
Largest diff. peak and hole	1.718 and -1.389 e.$Å^{-3}$	

Table S2. Crystal data and structure refinement for C2.

Identification code	**C2**	
Empirical formula	C81 H54 K O52 U6	
Formula weight	3326.52	
Temperature	100(2) K	
Wavelength	0.71073 Å	
Crystal system	Cubic	
Space group	I-43m	
Unit cell dimensions	a = 32.3525(15) Å	a= 90°.
	b = 32.3525(15) Å	b = 90°.
	c = 32.3525(15) Å	g = 90°.
Volume	33863(5) $Å^3$	
Z	8	
Density (calculated)	1.305 Mg/m^3	
Absorption coefficient	5.800 mm^{-1}	
F(000)	12216	
Crystal size	0.01 x 0.01 x 0.01 mm^3	
Theta range for data collection	0.890 to 24.725°.	
Index ranges	-26<=h<=26,-26<=k<=26,-37<=l<=37	
Reflections collected	20520	
Independent reflections	20520[R(int) = ?]	
Completeness to theta =24.725°	99.2 %	
Absorption correction	Empirical	
Max. and min. transmission	0.760 and 0.675	
Refinement method	Full-matrix least-squares on F^2	
Data / restraints / parameters	20520/ 103/ 239	
Goodness-of-fit on F^2	1.401	
Final R indices [I>2sigma(I)]	R1 = 0.0940, wR2 = 0.2260	
R indices (all data)	R1 = 0.2034, wR2 = 0.2798	
Flack parameter	x =-0.019(11)	
Largest diff. peak and hole	2.548 and -1.604 e.$Å^{-3}$	

Table S3. Crystal data and structure refinement for C3.

Identification code	**C3**	
Empirical formula	C254 H401 N14 O72 U8	
Formula weight	6707.10	
Temperature	100(2) K	
Wavelength	0.71073 Å	
Crystal system	Monoclinic	
Space group	P2(1)/n	
Unit cell dimensions	a = 19.8326(12) Å	a= 90°.
	b = 20.0478(12) Å	b = 95.583(2)°.
	c = 68.820(4) Å	g = 90°.
Volume	27233(3) Å3	
Z	4	
Density (calculated)	1.636 Mg/m^3	
Absorption coefficient	4.820 mm^{-1}	
F(000)	13340	
Crystal size	0.01 x 0.01 x 0.005 mm^3	
Theta range for data collection	0.595 to 27.901°.	
Index ranges	-26<=h<=18,-25<=k<=21,-59<=l<=90	
Reflections collected	188517	
Independent reflections	58942[R(int) = 0.0823]	
Completeness to theta =27.901°	90.5 %	
Absorption correction	Empirical	
Max. and min. transmission	0.795 and 0.577	
Refinement method	Full-matrix least-squares on F2	
Data / restraints / parameters	58942/ 5459/ 3311	
Goodness-of-fit on F2	1.196	
Final R indices [I>2sigma(I)]	R1 = 0.0839, wR2 = 0.1753	
R indices (all data)	R1 = 0.1150, wR2 = 0.1862	
Largest diff. peak and hole	3.067 and -1.546 e.$Å^{-3}$	

^{1}H and ^{13}C NMR spectra for 1

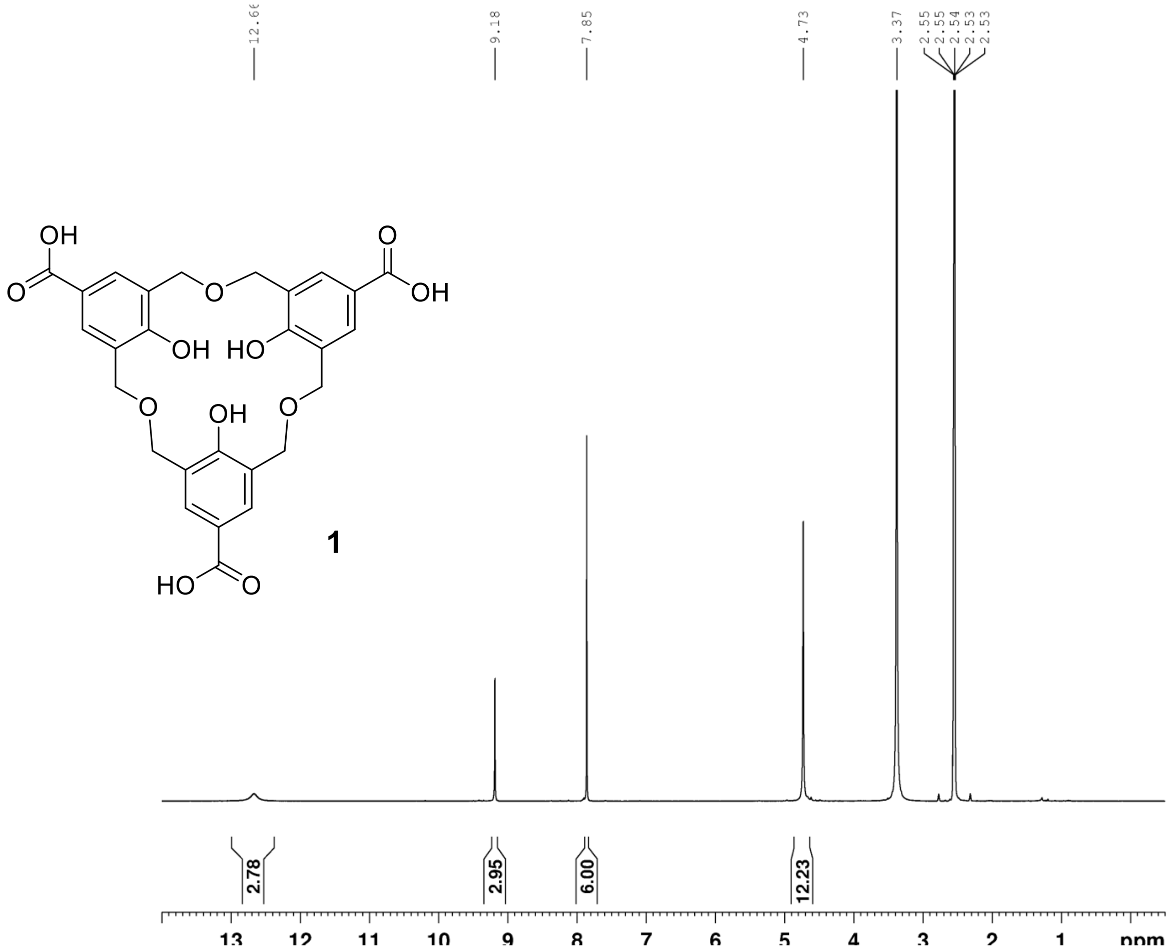

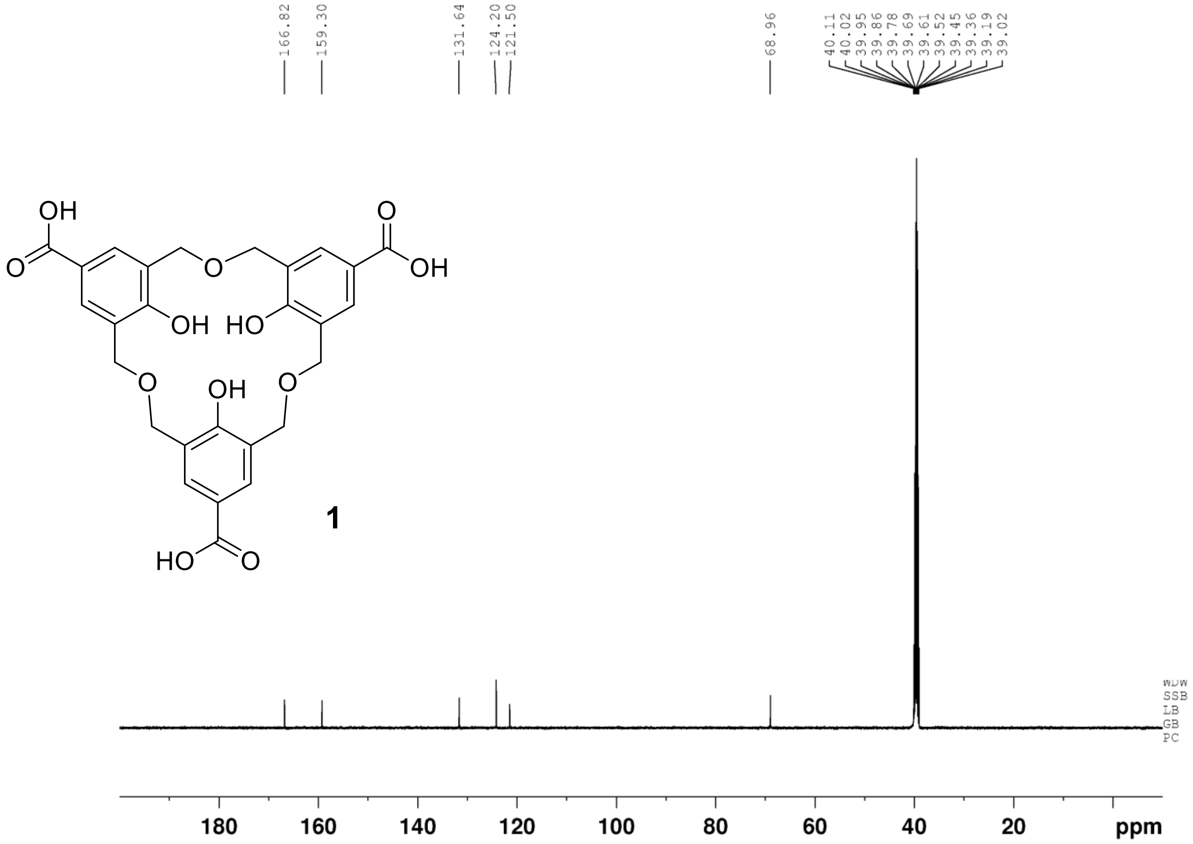
166.82
159.30
131.64
124.20
121.50
68.96
40.11
40.02
39.95
39.86
39.78
39.69
39.61
39.52
39.45
39.36
39.19
39.02
OH
O
OH
O
OH
HO
O
O
OH
HO
O
1
180
160
140
120
100
80
60
40
20
ppm
